# Searchable and revocable multi-data owner attribute-based encryption scheme with hidden policy in cloud storage

**DOI:** 10.1371/journal.pone.0206126

**Published:** 2018-11-01

**Authors:** Shangping Wang, Tingting Gao, Yaling Zhang

**Affiliations:** 1 School of Science, Xi’an University of Technology, Xi’an, Shaanxi, China; 2 School of Computer Science and Engineering, Xi’an University of Technology, Xi’an, Shaanxi, China; University of Colorado Denver, UNITED STATES

## Abstract

With the development of outsourcing data services, data security has become an urgent problem that needs to be solved. Attribute-based encryption is a valid solution to data security in cloud storage. There is no existing scheme that can guarantee the privacy of access structures and achieve attribute-based encryption with keyword search and attribute revocation. In this article, we propose a new searchable and revocable multi-data owner attribute-based encryption scheme with a hidden policy in cloud storage. In the new scheme, the same access policy is used in both the keyword index and message encryption. The advantage of keyword index with access policy is that as long as a user’s attributes satisfy the access policy, the searched ciphertext can be correctly decrypted. This property improves the accuracy of the search results. The hidden policy is used in both the ciphertext and the keyword index to protect users’ privacy. The new scheme contains attribute revocation, which is suitable for the actual situation that a user’s attributes maybe changed over time. In the general bilinear group model, the security of the scheme is demonstrated, and the efficiency of the scheme is analyzed.

## 1. Introduction

With technological developments, enterprise and personal data, photos, documents, and even health records maybe outsourced to cloud storage. Jiang D et al. [1] proposed a way to solve the network routing problem in cloud computing, it can achieve higher network energy efficiency for cloud computing. Siddiqui Z et al. [2] proposed in the dynamic cloud environment, the application of telemedicine information system provides convenience for patients and doctors. Along with the many benefits that cloud storage provides, it also presents serious data security problems. The data uploaded to the cloud should be encrypted to prevent information leakage. However, traditional encryption methods cannot be used to achieve access control and keyword searches. Therefore, we ask the following question. How can the data owners encrypt their data and enable both access control and quick searching in cloud storage?

Waqar A et al. [3] proposed a framework for preservation of cloud users' data privacy using dynamic reconstruction of metadata, it can protect the cloud users’ data privacy. Lin H Y et al. [4] proposed a scheme by use of threshold encryption and group signature mechanism to ensure the security of transmission data, it can ensure that the split and merged messages are not broken.

In traditional public-key cryptography, a message is encrypted for a specific receiver using the receiver’s public-key. Identity-based cryptography and in particular identity-based encryption (IBE) changed the traditional understanding of public-key cryptography by allowing the public-key to be an arbitrary string, e.g., the email address of the receiver. ABE goes one step further and defines the identity not atomic but as a set of attributes, e.g., roles, and messages can be encrypted with respect to subsets of attributes (key-policy ABE—KP-ABE) or policies defined over a set of attributes (ciphertext-policy ABE—CP-ABE). The key issue is, that someone should only be able to decrypt a ciphertext if the person holds a key for “matching attributes” (more below) where user keys are always issued by some trusted party. Attribute-based encryption technology can not only protect the privacy of data, but also solve the problem of information sharing in practical application. For attribute-based encryption scheme, data access control is an effective way to ensure data security. Attribute-based encryption enables fine-grained access control for data. A security issue in the cloud environment is the search problem. The data in cloud servers is stored in ciphertext, which guarantees the privacy of data. Once a user needs to find a relevant document containing a keyword, he will encounter the problem of how to search. The server performs a search operation, but does not know what the user is searching for. It can effectively protect the privacy of user search. Of course, in a cloud storage system, data access is not static. For example, if an employee is fired or promoted, the corresponding attribute needs to be changed. The attribute encryption technology supports multiple data owners to upload encrypted personal information records, and can conduct multiple keyword searches. It also allows data owners to search for different periods of time for multiple users.

In the existing attribute-based encryption schemes, the cloud server must know the accessing strategy to perform the keyword search operation. This requirement makes it a difficult task to simultaneously achieve searchability and protect the privacy of the access control. The hidden strategy can protect the privacy of the user's attributes, and the user's attributes may frequently change in practice. Therefore, the attribute revocation mechanism is essential. An attribute change for a single user may lead to changes of other users’ private keys that are associated with the attribute and even the changes of the ciphertext corresponding to the attribute.

How to structure a searchable and revocable attribute-based encryption scheme with hidden policy for multi-data owners in cloud storage is a challenging problem.

### 1.1 Advantages of the scheme

In this scheme, we proposed a searchable and revocable attribute-based encryption scheme with hidden policy for multi-data owners in cloud storage. The primary advantages of the scheme are summed up as follows:

- In our new scheme, the same access policy is used in message encryption and keyword index construction. The benefit of using the access policy in the construction of the keyword index is that as long as a user’s attributes satisfy the access structure, when the user submits a search token containing the secret attribute key to the search server, the search server can search the documents the user is interested in. The search results can then be decrypted by the user. Thus, the access policy is considered in the search process, which improves the accuracy of the search results.
- The access policy is hidden in the ciphertext and keyword index. The hidden policy can protect the privacy of the user's attributes.
- This scheme has the function of attribute revocation. If a user’s attribute changes, the index, ciphertext and private key connected with the attribute can be updated in time to ensure the security of the information.
- A search server is introduced in the system. It is used to store the keyword index. The ciphertext is stored in the cloud storage server. A keyword search is performed by the search server. For an authenticated user, the user gives the corresponding search token to a search server, and a search server responds to the search. When his attributes satisfy the access control structure and the given keyword is matched, a search server notifies the cloud storage server to send the relevant ciphertext to the user for decryption.
- In the general bilinear group model, it is demonstrated that the keyword index is secure under the keyword guessing attack and that the ciphertext is indistinguishable under the chosen-plaintext attack.

### 1.2 Related research

#### Attribute-based encryption (*ABE*)

Sahai and Waters [5] devised the first attribute-based encryption system, which was a historic breakthrough. Subsequently, Bethencourt, Sahai and Waters [6] in 2005 constructed an attribute encryption with ciphertext policy. The model can achieve fine-grained access control of ciphertext through attributes. There are two forms in the *ABE*: the ciphertext policy (*CP*−*ABE*) and the key policy (*KP*−*ABE)*. In the ciphertext policy, access control policy is embedded in the encrypted ciphertext, and the private key is related to attribute set. Only when the attributes of the user meet the ciphertext policy can the ciphertext can be decrypted. In the key policy, the ciphertext is related to the description of the attribute set, and the user's private key is related to the access structure. The access policy is defined on the attribute set. When the attribute sets meet the access control, the private key of the attributes can decrypt the corresponding ciphertext. Ling and Newport [7] proposed a provable secure ciphertext policy ABE, but the access structure in their scheme can only support the gate condition. However, none of these schemes supports keyword search ([8, 9, 10]).

#### Attribute-based encryption with keyword search (*ABKS*)

Song et al. [11] proposed the first initial searchable encryption program. In addition to the search results, the server knew nothing about the search keyword. Miao Y et al.[12] proposed a secure cryptographic primitive called as attribute-based multi-keyword search over encrypted personal health records in multi-owner setting to support both fine-grained access control and multi-keyword search via Ciphertext-Policy Attribute-Based Encryption. In [13], the authors propose a searchable encryption scheme for keywords in the hidden strategy. If the data user's attributes do not meet the access policy, the user cannot obtain the information of the access policy and cannot search for the encrypted data. Its innovation lies in the construction of the keyword index for the concealed access structure. However, there is no attribute-based encryption, and there is only a single data owner in the scheme in this article, while in practical situations, there should be multi-data owners in the system. Zheng and Sun ([14, 15]) in 2014 proposed two attribute-based keyword search schemes (*ABKS*). A data owner grants the search ability to users through the setup of an access policy, which effectively improves the search efficiency. The cloud server sends corresponding find results to the user when the user's attributes meet the access control structure, which is specified by data owner. Tang Y et al. [16] constructed a multi-keyword search scheme that applied to the network environment, based on privacy protection and efficiency. Xia Z [17] proposed multiple keyword searches and dynamic updates in cloud storage. Zhong H et al.[18] proposed a decentralized multi-authority CP-ABE access control scheme supporting the user revocation. Guo C et al. [19] constructed the access control of individual cases stored in the cloud server, it can achieve fine-grained access control for EHR. Also it allows multiple users to search on different databases. Fan Y et al. [20] constructed a verifiable scheme to support multi-keyword search. Guo Z [21] proposed a multi-keyword sorting search and supported the sharing of search functions. However, none of these existing schemes can hide access structures [22].

#### Attribute-based encryption hidden policy

Lai J et al. [23–26] provided some attribute-based hidden policy encryption schemes. In these schemes, access structure embedded in the ciphertext, for those attributes do not meet the access structure users cannot decrypt the ciphertext.

#### Attribute-based encryption revocation (ABER)

Tian et al. [27] proposed a revocable attribute-based encryption project. In this project, once a user is revoked, it is necessary to assign new keys to all other users in the system, except the revoked user. Then, a new encryption key re-encrypts the ciphertext. Thus, the revoked user can no longer decrypt the ciphertext. The method is not smart, because in practice the revoked users are only a small part and the majority of users are not revoked. Therefore, the method is infeasible. Zhihua Xia[28] presented an attribute-based access control project with valid revocation in cloud computing. The revocation is implemented using the version number of the private key, and the scheme also supports the backward security and forward security. The scheme is demonstrated to be effective and secure. Chen J [29] proposed an attribute-based encryption scheme with revocation and update in the cloud storage, in which the user is directly revoked. Li X et al. [30] proposed the revocation of two factors that are based on attribute encryption under the cloud storage, combining identity and attribute. Liu Z et al. [31] proposed a solution to update the user's access rights in a timely manner once the user's attributes change, and the data owner updates access control. In addition, there are some other articles that discuss the methods of attribute revocation for attribute-based encryption scheme ([32, 33, 34, 35, 36]).

## 2. Preliminaries

### 2.1 Bilinear map

Let $\left(e,p,g_{1},g_{2},G_{1},G_{2},G_{T}\right)\leftarrow BGenMap(1^{\lambda })$ be represented by a symmetric bilinear map $e:G_{1}\times G_{2}\to G_{T}$, where *λ* is a security parameter, $G_{1},G_{2},G_{T}$ are three multiplicative cyclic groups with the same order of prime *p*, and $g_{1}\in G_{1},g_{2}\in G_{2}$ are the generators of $G_{1,}G_{2}$, respectively. The bilinear *e* meets the following four conditions:

1. Bilinearity: $\forall \left(g_{1},g_{2}\right)\in G_{1}\times G_{2},\forall a,b\overset{R}{\leftarrow }{\mathbb{Z}}_{p}:e\left(g_{1}^{a},g_{2}^{b}\right)=e{\left(g_{1,}g_{2}\right)}^{ab}$;
2. Non-degeneracy: *e*(*g*_1_,*g*_2_) ≠ 1;
3. Efficiency: There is a valid polynomial time algorithm to compute *e*(*g*_1_,*g*_2_), $\forall \left(g_{1},g_{2}\right)\in G_{1}\times G_{2}$;
4. There is a valid, publicly calculated (no need reversible) isomorphism $\psi :G_{2}\to G_{1}$ such that *ψ*(*g*_2_) = *g*_1_.

### 2.2 Generic bilinear group model

Let $\left(e,p,g_{1},g_{2},G_{1},G_{2},G_{T}\right)\leftarrow BGenMap(1^{\lambda })$ be defined as follows. In a general linear group model, three random codes are assumed as $\varsigma_{1},\varsigma_{2},\varsigma_{T}:{\mathbb{Z}}_{p}^{+}\to {\left\{0,1\right\}}^{m}$. ${\mathbb{Z}}_{p}^{+}$ is an addition group, and *m*>3log*p*. For *i* = 1,2,*T*, let $G_{i}=\left\{\varsigma_{i}\left(x\right)|x\in {\mathbb{Z}}_{p}^{+}\right\}$. There are three oracles to compute the operation in the groups $G_{1},G_{2},G_{T}$. There are oracles to compute non-degenerate linear maps $e:G_{1}\times G_{2}\to G_{T}$.

### 2.3 Access structure

Definition 1. Index *n* attributes in the system can be denoted as *U* = {1,2,⋯,*n*}. For each attribute *i*∈*U*,let $S_{i}=\left\{v_{i,1},v_{i,2}\cdots v_{i,n_{i}}\right\}$ denote all the possible values for this attribute, where *n*_*i*_ is the number of possible values for this attribute *i*. Suppose that *L* = {*L*_1_,*L*_2_⋯*L*_*n*_} is a list of a user’s attributes, where *L*_*i*_∈*S*_*i*_. *P* = {*P*_1_,*P*_2_⋯*P*_*n*_} is an access structure, where *P*_*i*_⊆*S*_*i*_. Define *L*| = *P* if the attribute list *L* = {*L*_1_,*L*_2_⋯*L*_*n*_} meets the access structure *P* = {*P*_1_,*P*_2_⋯*P*_*n*_}. In other words *L*_*i*_∈*S*_*i*_, for all *i*,1≤*i*≤*n*.

## System model and security model

### 3.1 System entities

**Fig 1:**
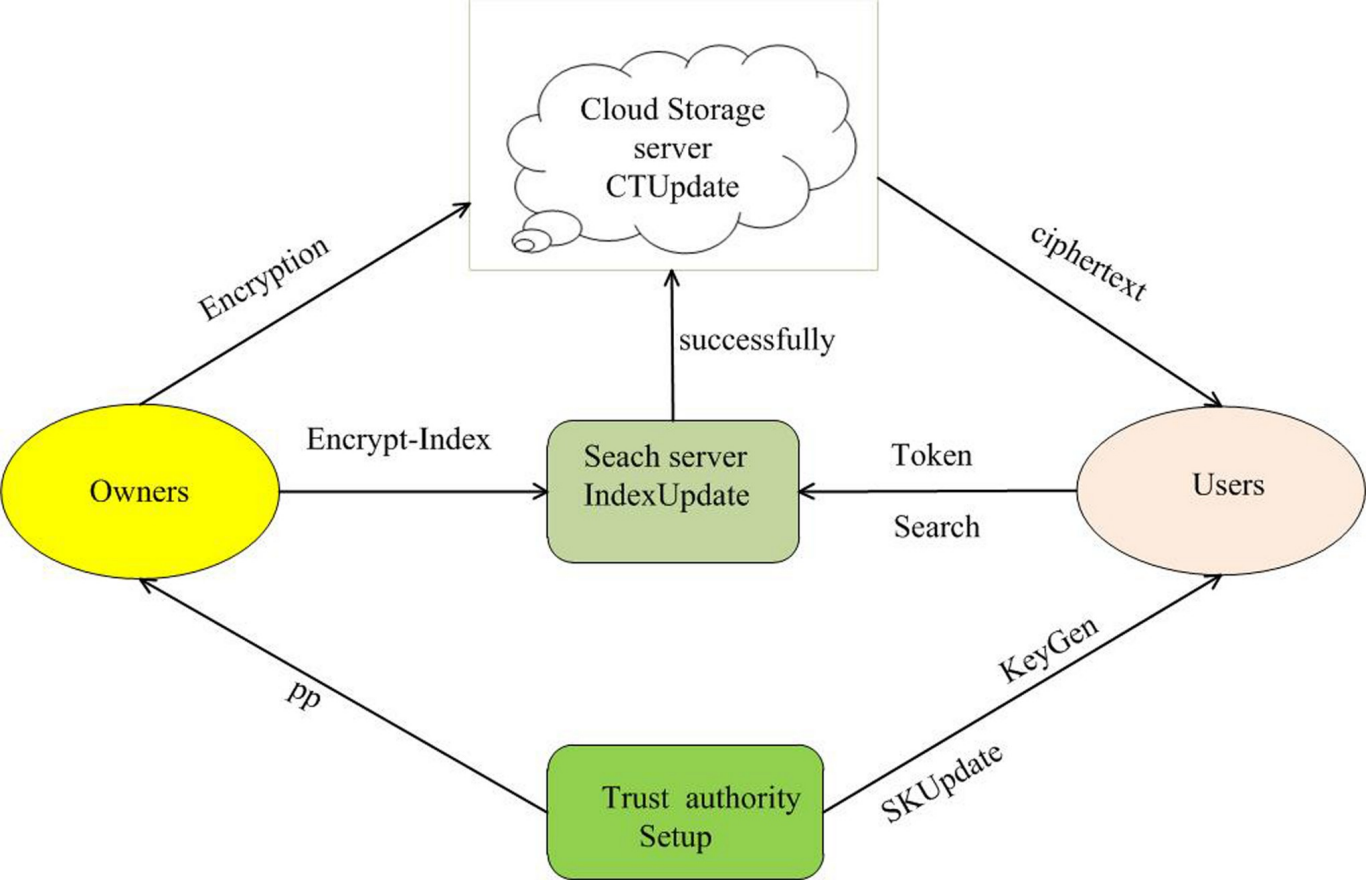
System model.

Above all, the system framework is described in Fig 1.The framework includes the five main entities of the trusted authority, the cloud storage server, the search server, the multi-data owners, and multi-users. Particularly, the trusted authority controls the common parameters and distributes certified users’ private keys. The private key is related with the user's attribute list. The cloud storage server provides storage capabilities. Data owners encrypt messages, construct keyword indexes. Owners outsource encrypted messages to the cloud. The keyword index is outsourced to the search server, and the search server is responsible for matching. A certified data user in the system can generate a keyword search token related to his attributes’ private key. A search token is presented to a search server, and the search server searches for the keyword index. If the user’s attribute list meets the access structure implied in the keyword index, the successful search is returned to a cloud server. Next, the cloud storage server sends the ciphertext corresponding to the keyword index to the user.

### 3.2 Function definition

Definition 2: Our scheme contains a set of polynomial time algorithms

$$\Pi =\left(\begin{array}{l} Setup,KeyGen,Encrypt,Encrypt-Index,GenToken,\\ Search,Decrypt,SKUpdate,CTUpdate,IndexUpdate \end{array}\right)$$ (1)

as described below.

Setup (1^*λ*^)→(*msk*,*pp*): The algorithm is executed by a trusted authentication attribute authority. It takes the security parameter *λ* as an input and outputs the public parameter *pp* and the master key *msk*.

KeyGen (*msk*,*pp*,*L*)→*sk*: The algorithm is used to produce a user's private key by a trusted authority. It takes the master key *msk*, the public parameter *pp*,and the users attribute list *L* as the inputs. It outputs the key *sk* associated with *L*.

Encryption (*pp*,*m*,*P*)→*ct*: A data owner executes the algorithm. It takes the common parameters *pp*,a message *m*, and one access control *p* as inputs. It outputs a ciphertex *ct*.

Encrypt-Index (*pp*,*w*,*P*)→*Index*: A data owner executes the algorithm. It takes the common parameters *pp*, a set of keywords *w*, and an access control structure *p* as inputs. It then exports the key *Index*.

GenToken (*sk*,*w*)→*tok*: A data user executes the algorithm to produce search tokens for queries. It takes the input private key *sk* and a keyword *w* as inputs. It then exports the keyword search token *tok*.

Search (*tok*,*Index*)→{0,1}: A search server runs the algorithm. It takes the keyword index *Index*←(*pp*,*w*,*P*) and a search token *tok*←(*sk*,*w*′) as inputs. It then outputs 1 if *L*↦*P* and *w* = *w*′. Otherwise, it outputs 0.

Decryption (*CT*,*sk*)→*m*: A data user executes the decryption algorithm. It takes the ciphertext *ct* and the decryption key *sk* as inputs and outputs message *m*.

CTUpdate $\left({\hat{c}}_{i,j,2},u_{i,j}\right)\to {\hat{c}}_{i,j,2}^{\prime }$, The cloud storage server executes the ciphertext update algorithm. It takes ciphertext *ct* and update operator (*v*_*i*,*j*_,*u*_*i*,*j*_) as inputs. It then outputs the updated ciphertext *ct*′.

SKUpdate $\left(K_{i,t_{i},1},u_{i,j}\right)\to K_{i,t_{i},1}^{\prime }$: The authority executes the user's private key update algorithm. It takes the user's private key $K_{i,t_{i},1}$ and the update operator (*v*_*i*,*j*_,*u*_*i*,*j*_) as inputs. It then outputs the updated key *sk*′.

IndexUpdate $\left(I_{i,j,2},u_{i,j}\right)\to I_{i,j,2}^{\prime }$:The search server executes the update index algorithm. It takes the update operator (*v*_*i*,*j*_,*u*_*i*,*j*_)as an input and then exports the updated index *Index*′.

### 3.3 Security definition

1.The secure game of indistinguishability of keyword index under the selective keyword attack with hidden policy.

System establishment: The adversary selects two access control strategies *P*_0_,*P*_1_.He needs to send them to the challenger. The challenger selects safety parameters *λ* and runs the Setup(*λ*) algorithm, generating public parameters *pp* and mask secret key *msk*.The challenger gives the public parameter *pp* to the adversary and leaves the master secret key *msk*.

Phase 1. The adversary selects a list of attributes *L* such that *L*| ≠ *P*_0_∧*L*| ≠ *P*_1_. He then asks in polynomials times as follows:

*O*_*KeyGen*_(*L*). The challenger generates the private key *sk* though KeyGen (*msk*,*pp*,*L*)→*sk* and gives it to the adversary.

*O*_*GenToken*_(*L*,*w*). The challenger generates *sk* through *O*_*KenGen*_(*L*). He then runs the token-generating algorithm GenToken (*sk*,*w*)→*tok* to get the token and return it to the adversary.

Challenge. The adversary submits two challenge keywords *w*_0_,*w*_1_ to the challenger. The condition is that the adversary has not asked for any search tokens of *w*_0_,*w*_1_. The challenger chooses a random bit *b*∈{0,1}. He then produces the index *I*_*b*_ by the index generating algorithm for keyword *w*_*b*_ under policy *P*_*b*_, and returns the index *I*_*b*_ to the adversary A.

Phase 2. The adversary A can still query similar to Phase 1.The restricted condition is *w* ≠ *w*_0_,*w*_1_.

Guess: The adversary A outputs a guess *b*′ for *b*. If *b*′ = *b*, then the adversary wins this game.

The adversary's advantage in the game is defined as

$$Adv\left(A\right)=\left|\mathrm{Pr}\left[b^{\prime }=b\right]-1/2\right|$$ (2)

If there is no polynomial time, the adversary can win the above game with a non-negligible advantage. Next, the scheme is called secure in the sense of the indistinguishability of keyword index under the selective keyword attack with the hidden policy.

2. The secure game of indistinguishability of ciphertext under the selective plain-text attack with the hidden policy.

System establishment. The adversary selects two access control strategies *P*_0_,*P*_1_. He then transmits them to a challenger. The challenger selects the safety parameter *λ* and runs the Setup(*λ*) algorithm, generating public parameters *pp* and master secret key *msk*.The challenger gives the public parameter *pp* to the adversary and later leaves the master secret key *msk*.

Phase 1. The adversary selects a list of attributes *L* such that *L*| ≠ *P*_0_∧*L*| ≠ *P*_1_ and asks in polynomials times as follows *O*_*KeyGen*_(*L*).The challenger generates the private key *sk* though KeyGen (*msk*,*pp*,*L*)→*sk* and provides it to the adversary.

Challenge. The adversary submits two equal length messages *m*_0_,*m*_1_ to the challenger. The challenger selects a random bit *b*∈{0,1}. He subsequently generates the ciphertext *ct*_*b*_ by the encryption algorithm for message *m*_*b*_ under policy *P*_*b*_ and returns the ciphertext *ct*_*b*_ to the adversary A.

Phase 2. The adversary A can still query similar as Phase 1.

Guess. The adversary A exports a guess *b*′ for *b*. If *b*′ = *b*, then he wins this game. The adversary's advantage in the game is defined as

$$Adv\left(A\right)=\left|\mathrm{Pr}\left[b^{\prime }=b\right]-1/2\right|$$ (3)

If there is no polynomial time adversary that can win the above game with a non-negligible advantage, then the scheme is called the secure model of indistinguishability of ciphertext under the selective plain-text attack with hidden policy.

## 4. Scheme construction

In this part, we will propose a searchable and revocable attribute-based encryption scheme with a hidden policy in cloud storage. Our new scheme achieves encryption and keyword search and attribute revocation. The access control policy consists of a set of *AND* gates. In the system we assume that there are *n* attributes, and all the attributes are labeled as {1,2,⋯*n*}.

### 4.1 Motivation

Authors in [13] proposed a searchable encryption scheme with the hidden strategy, and the hidden strategy is a major feature of the scheme. However, we find that the scheme is correct only if the data owner and the data user are the same one, this error is not easy to find as they use the same parameter *r* for the data owner and data user. Here we will give a detail analysis, in order to demonstrate this problem, we use different parameter *r* for the data owner and data user.

The data owner’s parameters can be denoted by $X_{0}=Y^{x_{o}},C_{u_{0}}=X_{0}^{-r_{0}}(x_{0}\in {\mathbb{Z}}_{p}),{\tilde{C}}_{0}=Y^{r_{0}}.$ in which *r*_0_ is randomly selected by the data owner, *x*_0_ is randomly selected by attribute authority for the data owner. Data user’s parameters denoted by $X_{u}=Y^{x_{u}},C_{u_{u}}=X_{u}^{-r_{u}}(x_{u}\in {\mathbb{Z}}_{p}),\tilde{T}=x_{u}+s$,in which *r*_*u*_ is randomly selected by the data user, *x*_*u*_ is randomly selected by attribute authority for the data user. In the search algorithm, the match equation should be ${\tilde{C}}^{\tilde{T}}\cdot C_{u_{u}}=Y^{r_{0}s}=e{\left(g_{1},g_{2}\right)}^{r_{0}s\alpha }$, but if the data owner and the data user are not the same one, we find that ${\tilde{C}}^{\tilde{T}}\cdot C_{u_{u}}={\tilde{C}}^{\left(x_{u}+s\right)}\cdot X_{u}^{-r_{u}}=Y^{r_{0}\left(x_{u}+s\right)}\cdot Y^{-x_{u}r_{u}}=Y^{x_{u}\left(r_{0}-r_{u}\right)}\cdot Y^{r_{0}s}$, which is not equal to $Y^{r_{0}s}$ except for *r*_0_ = *r*_*u*_. Thus the original scheme is correct only for the data owner and the data user are same one. This limits the usefulness of the scheme in [13].

To overcome these problems, we improve the scheme in reference [13].We will take the public parameter *r* and design a new scheme that accounts for multiple data owners. Another improvement of our new scheme is to simplify the hash function with a key [13] to a general hash function without a key to increase the practicality of the scheme. Since all users in the system share a secret key for the hash function, it is actually not secure, and the server can easily collude with a user to get the key. We also add attribute encryption and attribute revocation to make the scheme feasible and retain the advantages of the hidden access structure.

### 4.2 Our construction

The scheme consists of the following algorithms.

- *Setup*(1^*λ*^): Input the security parameter *λ*. Then, the algorithm produces the public parameters and the master secret key as follows:
  1. $Generate\left(1^{\lambda }\right)\to \left(e,p,g_{1},g_{2},G_{1},G_{2},G_{T},H\right)$, where *e* is a symmetric bilinear mapping *e*:$G_{1}\times G_{2}\to G_{T}$. *g*_1_,*g*_2_ are the generators of $G_{1,}G_{2}$, respectively. $G_{1},G_{2},G_{T}$ are three multiplicative cyclic groups of prime orders *p*. $H:{\left\{0,1\right\}}^{*}\to {\mathbb{Z}}_{p}$ is a secure hash function.
  2. For each attribute *i*,1≤*i*≤*n*. Let $S_{i}=\left\{v_{i,1},v_{i,2}\cdots v_{i,n_{i}}\right\}$ be a set of all possible values of attribute *i*. It generates a random values set ${\left\{a_{i,j}\in {\mathbb{Z}}_{p}\right\}}_{1\leq j\leq n_{i}}$ for attribute *i* and calculates ${\left\{A_{i,j}=g_{1}^{a_{i,j}}\right\}}_{1\leq j\leq n_{i}}$.It chooses $\alpha ,\beta ,b\overset{R}{\leftarrow }{\mathbb{Z}}_{p}$, and calculates $Y=e{\left(g_{1},g_{2}\right)}^{\alpha },B=g_{1}^{b}$, and $K_{0}=g_{2}^{\frac{\alpha +\beta }{b}}$. It chooses a random number $r\overset{R}{\leftarrow }{\mathbb{Z}}_{p}$ and publishes it and then calculates $\hat{I}=Y^{r}$, *I*_0_ = *B*^*r*^. The public parameter *pp* and the master secret key *msk* are set as follows:
    $$pp=\left(e,p,g_{1},g_{2},g_{2}^{\beta },G_{1},G_{2},G_{T},H,Y,B,K_{0},{\left\{{\left\{A_{i,j}\right\}}_{1\leq j\leq n_{i}}\right\}}_{1\leq i\leq n},r,\hat{I},I_{0}\right)$$ (4)
    $$msk=\left(\alpha ,\beta ,b,{\left\{{\left\{a_{i,j}\right\}}_{1\leq j\leq n_{i}}\right\}}_{1\leq i\leq n}\right).$$ (5)
- *KeyGen*(*msk*,*pp*,*L*):Suppose $L=\left\{L_{1},L_{2},\cdots L_{n}\right\}=\left\{v_{1,t_{1}},v_{2,t_{2}},\cdots v_{n,t_{n}}\right\}$ is the attribute list of a user *U*. User *U* chooses one of his own $x_{u}\overset{R}{\leftarrow }{\mathbb{Z}}_{p}$, calculates $X_{u}=Y^{x_{u}}$, and then submits it to the attribute authority. The user saves *x*_*u*_.Next, for each attribute *i*,1≤*i*≤*n*, the authority chooses $\lambda_{i}\overset{R}{\leftarrow }{\mathbb{Z}}_{p}$ and calculates $K_{i,t_{i},1}=g_{2}^{\beta +a_{i,t_{i}}\lambda_{i}},K_{i,2}=g_{2}^{\lambda_{i}}$. It finally sets the private key of the user with the attribute list $L=\left\{L_{1},L_{2},\cdots L_{n}\right\}=\left\{v_{1,t_{1}},v_{2,t_{2}},\cdots v_{n,t_{n}}\right\}$ as follows:
  $$sk=\left(x_{u},{\left\{K_{i,t_{i},1},K_{i,2}\right\}}_{1\leq i\leq n}\right).$$ (6)
  The authority add tuples (*U*,*I*_*u*_,*L*) to the list of users *U*_*List*_, where *I*_*u*_ = *X*_*u*_^−*r*^. Next, the authority sends *U*_*List*_ to the search server.
- *Encrypt*(*pp*,*m*,*P*):Suppose *P* = {*P*_1_,*P*_2_,⋯*P*_*n*_} is an access control policy, *P*_*i*_⊆*S*_*i*_. When outsourcing a file *F* to a cloud storage server, the algorithm produces a ciphertext that is related to the access control structure *P* as follows:
  1. Above all randomly chooses $\hat{r}\overset{R}{\leftarrow }{\mathbb{Z}}_{p}$, $\hat{c}=me{\left(g_{1},g_{2}\right)}^{\alpha \hat{r}}$, and ${\hat{c}}_{0}=B^{\hat{r}}$,where *m* is the key to encrypt the file *F* by a symmetric encryption algorithm.
  2. For each *i*,1≤*i*≤*n*, it first selects ${\hat{r}}_{i}\overset{R}{\leftarrow }{\mathbb{Z}}_{p}$ such that $\hat{r}={\sum_{i=1}^{n}\hat{r}}_{i}$ and it calculates ${\hat{c}}_{i,1}=g_{1}^{{\hat{r}}_{i}}$. For all *v*_*i*,*j*_∈*S*_*i*_, if *v*_*i*,*j*_∈*P*_*i*_, set ${\hat{c}}_{i,j,2}=A_{{}_{i,j}}^{{\hat{r}}_{i}}$.If *v*_*i*,*j*_∉*P*_*i*_, it sets ${\hat{c}}_{i,j,2}$ to a random value within $G_{1}$. It sets the ciphertext as
    $$CT=\left(\hat{c},{\hat{c}}_{0},{\left\{{\hat{c}}_{i,1},{\left\{{\hat{c}}_{i,j,2}\right\}}_{1\leq j\leq n_{i}}\right\}}_{1\leq i\leq n}\right).$$ (7)
- *Encrypt*−*Index*(*pp*,*w*,*P*):Suppose *P* = {*P*_1_,*P*_2_,⋯*P*_*n*_} is an access control policy, which is the same access control policy as it is in the encrypted ciphertext. Let *w* be a keyword extracted from file *F*. It produces a secure keyword index related to the access control policy *P* as follows:

For each attribute *i*,1≤*i*≤*n*, above all selects $r_{i}\overset{R}{\leftarrow }{\mathbb{Z}}_{p}$, makes $r=\sum_{i=1}^{n}r_{i}$, and computes $I_{i,1}=g_{1}^{r_{i}}$. For all *v*_*i*,*j*_∈*S*_*i*_, if *v*_*i*,*j*_∈*P*_*i*_, $I_{i,j,2}=A_{{}_{i,j}}^{r_{i}/H\left(w\right)}$ is set. If *v*_*i*,*j*_∉*P*_*i*_, *I*_*i*,*j*,2_ is set to a random value in $G_{1}$. The keyword index of the keyword *w* is

$$Index=\left({\left\{I_{i,1},{\left\{I_{i,j,2}\right\}}_{1\leq j\leq n_{i}}\right\}}_{1\leq i\leq n}\right).$$ (8)

If the file has multiple keywords, it can be used to generate multiple security indexes.

Note that here *r* is the public system parameter and is the same for all keyword index generations.

- $GenToken\left(sk,\tilde{w}\right)$: This algorithm generates a secure search token for a keyword $\tilde{w}$. If a user *U* wants to search a keyword $\tilde{w}$,the user *U* chooses a $s\overset{R}{\leftarrow }{\mathbb{Z}}_{p}$ and then computes $t=x_{u}+s,T_{0}=K_{0}^{s}$ for each *i*,1≤*i*≤*n*. It computes $T_{i,t_{i},1}=K_{i,t_{i},1}^{s},T_{i,2}=K_{i,2}^{H\left(\tilde{w}\right)s}$. Finally, it sets the search Token as
  $$tok=\left(t,T_{0},{\left\{T_{i,t_{i},1},T_{i,2}\right\}}_{1\leq i\leq n},U\right).$$ (9)
- *Search*(*tok*,*Index*): Once the token of user *U* is received, the search server first checks whether the *U* is in *U*_*List*_. If it is not, the request s refused. Otherwise, it gets the tuples (*U*,*I*_*u*_,*L*),where $L=\left\{L_{1},L_{2},\cdots L_{n}\right\}=\left\{v_{1,t_{1}},v_{2,t_{2}},\cdots v_{n,t_{n}}\right\}$ and $I_{u}=X_{u}^{-r}$.The search server runs the matching algorithm for (*tok*,*Index*) and (*U*,*I*_*u*_,*L*) as follows:
  1. It computes $E_{1}=\prod_{i=1}^{n}e\left(I_{i,1},T_{i,t_{i},1}\right)$.
  2. For each *i*,1≤*i*≤*n*, if $L_{i}=v_{i,t_{i}}$, it chooses $I_{i,t_{i},2}$ and computes $E_{2}=\prod_{i=1}^{n}e\left(I_{i,t_{i},2},T_{i,2}\right)$ and *E* = *E*_1_/*E*_2_ = *e*(*g*_1_,*g*_2_)^*srβ*^. If $e\left(I_{0},T_{0}\right)\cdot E^{-1}={\hat{I}}^{t}\cdot I_{u}$, the match is successful and returns 1.A notification is sent to the cloud server. The cloud server sends the corresponding ciphertext associated with the index to the user. Otherwise, the match is failed and returns 0.
- *Decrypt*(*CT*,*sk*): The decryption algorithm is run by data user *U* with attribute list $L=\left\{L_{1},L_{2},\cdots L_{n}\right\}=\left\{v_{1,t_{1}},v_{2,t_{2}},\cdots v_{n,t_{n}}\right\}$ to decrypt the ciphertext *CT* by using its secret key *sk*. For each *i*,1≤*i*≤*n* and $L_{i}=v_{i,t_{i}}$, it chooses ${\hat{c}}_{i,t_{i},2}$ and computes $\hat{E}=\prod_{i=1}^{n}e\left({\hat{c}}_{i,1},K_{i,t_{i},1}\right)/\prod_{i=1}^{n}e\left({\hat{c}}_{i,t_{i},2},K_{i,2}\right)$,
  $$m=\frac{\hat{E}\hat{c}}{e\left({\hat{c}}_{o},K_{0}\right)}.$$ (10)
- *AttriUpdate*(*v*_*i*,*j*_,*a*_*i*,*j*_): When a user's attribute *i* is revoked, suppose that the attribute value revoked is *v*_*i*,*j*_.The authority runs this update algorithm and selects a new random value $a_{i,j}^{\prime }$ instead of the old secret value *a*_*i*,*j*_ corresponding to *v*_*i*,*j*_. It publishes an attribute update operator (*v*_*i*,*j*_,*u*_*i*,*j*_), where $u_{i,j}=a_{i,j}^{\prime }/a_{i,j}\mathrm{mod}p$.
- *PPUpdate*(*A*_*i*,*j*_,*u*_*i*,*j*_): The authority inputs the update operator (*v*_*i*,*j*_,*u*_*i*,*j*_) and recalculates $A_{i,j}^{\prime }=A_{i,j}^{u_{i,j}}$ as the new system parameter to instead of the old parameter *A*_*i*,*j*_ for attribute *i* and publishes it in the system parameter set.
- $CTUpdate\left({\hat{c}}_{i,j,2},u_{i,j}\right)$: The ciphertext update algorithm inputs the ciphertext ${\hat{c}}_{i,j,2}$ and the update operator (*v*_*i*,*j*_,*u*_*i*,*j*_). It then outputs the new ciphertext ${\hat{c}}_{i,j,2}$ such that
  $${\hat{c}}_{i,j,2}^{\prime }={\left({\hat{c}}_{i,j,2}\right)}^{u_{i,j}}=g_{1}^{r_{i}a_{i,j}^{\prime }}.$$ (11)
- *SKUpdate*(*K*_*i*,1_,*u*_*i*,*j*_): The private key update algorithm inputs the private key *K*_*i*,1_ corresponding to attribute *v*_*i*,*j*_ and the update operator (*v*_*i*,*j*_,*u*_*i*,*j*_). It then outputs the new private key $K_{i,t_{i},1}^{\prime }$ as
  $$K_{i,t_{i},1}^{\prime }={\left(K_{i,t_{i},1}/g_{2}^{\beta }\right)}^{\lambda_{i}u_{i,j}}\cdot g_{2}^{\beta }=g_{2}^{\beta +\lambda_{i}a_{i,t_{i}^{\prime }}}.$$ (12)
- *IndexUpdate*(*I*_*i*,*j*,2_,*u*_*i*,*j*_): The index update algorithm inputs *I*_*i*,*j*,2_ (which is part of the index related to attribute *v*_*i*,*j*_) and the update operator (*v*_*i*,*j*_,*u*_*i*,*j*_). It then outputs the new index $I_{i,j,2}^{\prime }$ as
  $$I_{i,j,2}^{\prime }={\left(I_{i,j,2}\right)}^{u_{i,j}}={\left(g_{1}^{a_{i,j}r_{i}/H\left(w\right)}\right)}^{a_{i,j}^{\prime }/a_{i,j}}=g_{1}^{r_{i}a_{i,j}^{\prime }/H\left(w\right)}.$$ (13)

## 5. Security analysis

The correctness of algorithm *Search*(*tok*,*Index*) is as follows:

If *L*| = *P*, *w* = *w*′, and $L=\left\{L_{1},L_{2},\cdots L_{n}\right\}=\left\{v_{1,t_{1}},v_{2,t_{2}},\cdots v_{n,t_{n}}\right\}$,

$$E=\frac{E_{1}}{E_{2}}=\frac{\prod_{i=1}^{n}e\left(I_{i,1},T_{i,t_{i},1}\right)}{\prod_{i=1}^{n}e\left(I_{i,t_{i},2},T_{i,2}\right)}=\frac{\prod_{i=1}^{n}e\left(g_{1}^{r_{i}},g_{2}^{\left(\beta +a_{i,t_{i}}\lambda_{i}\right)s}\right)}{\prod_{i=1}^{n}e\left(g_{1}^{a_{i,j,2}r_{i}\frac{1}{H\left(w\right)}},g_{2}^{\lambda_{i}H\left(\tilde{w}\right)s}\right)}=\frac{\prod_{i=1}^{n}e{\left(g_{1},g_{2}\right)}^{sr_{i}\left(\beta +a_{i,t_{i}}\lambda_{i}\right)}}{\prod_{i=1}^{n}e{\left(g_{1},g_{2}\right)}^{sr_{i}\lambda_{i}a_{i,j,2}}}=\prod_{i=1}^{n}e{\left(g_{1},g_{2}\right)}^{sr_{i}\beta }=e{\left(g_{1}.g_{2}\right)}^{sr\beta },$$

If $e\left(I_{0},T_{0}\right)\cdot E^{-1}=e\left(B^{r},K_{0}^{s}\right)\cdot E^{-1}=e\left(g_{1}^{br},g_{2}^{\frac{\alpha +\beta }{b}s}\right)\cdot e{\left(g_{1},g_{2}\right)}^{-sr\beta }=e{\left(g_{1},g_{2}\right)}^{rs\alpha }$ is equal to ${\hat{I}}^{t}\cdot I_{u}=Y^{rt}\cdot X_{u}^{-r}=e{\left(g_{1},g_{2}\right)}^{\alpha r\left(x_{u}+s\right)}\cdot e{\left(g_{1},g_{2}\right)}^{-\alpha x_{u}r}=e{\left(g_{1},g_{2}\right)}^{rs\alpha }$, the match is successful and the search server returns 1.

The correctness of the decryption algorithm is verified as follows:

If *L*| = *P* and $L=\left\{L_{1},L_{2},\cdots L_{n}\right\}=\left\{v_{1,t_{1}},v_{2,t_{2}},\cdots v_{n,t_{n}}\right\}$ then

$$\hat{E}=\frac{\prod_{i=1}^{n}e\left({\hat{c}}_{i,1},K_{i,t_{i},1}\right)}{\prod_{i=1}^{n}e\left({\hat{c}}_{i,t_{i},2},K_{i,2}\right)}=\frac{\prod_{i=1}^{n}e\left(g_{1}^{{\hat{r}}_{i}},g_{2}^{\beta +a_{i,t_{i}}\lambda_{i}}\right)}{\prod_{i=1}^{n}e\left(g_{1}^{a_{i,j}{\hat{r}}_{i}},g_{2}^{\lambda_{i}}\right)}={\prod_{i=1}^{n}e\left(g_{1},g_{2}\right)}^{\beta {\hat{r}}_{i}}=e{\left(g_{1},g_{2}\right)}^{\beta \hat{r}}$$ (14)

$$m=\frac{\hat{E}\hat{c}}{e\left({\hat{c}}_{0},K_{0}\right)}=\frac{e{\left(g_{1},g_{2}\right)}^{\beta \hat{r}}\;me\;{\left(g_{1},g_{2}\right)}^{\alpha \hat{r}}}{e\left(g_{1}^{b\hat{r}},g_{2}^{\frac{\alpha +\beta }{b}}\right)}$$ (15)

Our safety analysis scheme is as follows:

We will analyze and demonstrate the security of our scheme under the general bilinear mapping model ([6, 14, 36]). First, we will prove that our scheme is of the indistinguishability of the keyword index under the selective keyword attack with the hidden policy. Second, we will demonstrate that our scheme is of the indistinguishability of the ciphertext under the selective plain-text attack with the hidden policy.

**Theorem 1**. Let $\varsigma_{1},\varsigma_{2},\varsigma_{T},G_{1},G_{2},G_{T}$ be defined as the general bilinear group model. We request that any adversary A performs up to *q* times oracles to ask for group $G_{1},G_{2},G_{T}$‘s calculation, including bilinear mapping. In the secure game of the indistinguishability of the keyword index under the selective keyword attack with hidden policy, the advantage of an adversary A is *O*(*q*^2^/*p*).

Proof: Our proof is similar to [13,24].We will design a simulator B and an adversary A to perform the indistinguishability of the keyword index under the selective keyword attack with the hidden policy as follows. A maintains 3 pairs of lists:

$$\begin{array}{l} V_{G_{1}}=\left\{\langle F_{1,l},\varsigma_{1,l}\rangle :l=1,\cdots ,\tau_{1}\right\},\\ V_{G_{2}}=\left\{\langle F_{2,l},\varsigma_{2,l}\rangle :l=1,\cdots ,\tau_{2}\right\},\\ V_{G_{T}}=\left\{\langle F_{T,l},\varsigma_{T,l}\rangle :l=1,\cdots ,\tau_{T}\right\}{}_{{}^{\circ}} \end{array}$$ (16)

In these equations, *F*_*τ*,*l*_(*τ*∈{1,2,*T*}) is adversary A's queries, *ς*_*τ*,*l*_(*τ*∈{1,2,*T*}) is a random string of {0,1}* for each query result, and *ς*_1,*l*_ = *ς*_1_(*F*_1,*l*_),*ς*_2,*l*_ = *ς*_2_(*F*_2,*l*_),*ς*_*T*,*l*_ = *ς*_*T*_(*F*_*T*,*l*_).

The initialization definition is *F*_1,1_ = 1,*F*_2,1_ = 1,*F*_*T*,1_ = 1, and *ς*_1,1_,*ς*_2,1_,*ς*_*T*,1_ is the initial mapping string. *ς*_1_(1) represents *g*_1_, *ς*_2_(1) represents *g*_2_, and *ς*_*T*_(1) represents *e*(*g*_1_,*g*_2_). In the following query, the adversary A and the simulator B use *ς* to represent the elements in the group. In particular, for each query, the simulator selects random real values contained in the list. Whenever A gives a query to B, B will update its list and return to the relevant random string to A. Next, we give A’s query as follows:

**Group action.** Set two operand objects *ς*_*τ*_(*x*),*ς*_*τ*_(*y*). Additionally, $x,y\overset{R}{\leftarrow }{\mathbb{Z}}_{p},\tau \in \left\{1,2,T\right\}$. If *ς*_*τ*_(*x*),*ς*_*τ*_(*y*) are not in the list $V_{G_{T}}$, they are returned. Otherwise, B computes *F* = *x*+*y* mod *p* and checks where *F* is in the list $V_{G_{T}}^{}$.If it is in it, it returns *ς*_*τ*_(*F*). Otherwise, B sets a random string in {0,1}* different from the list $V_{G_{T}}$ already exists in. Finally, B will be added 〈*F*,*ς*_*τ*_(*F*)〉 to the $V_{G_{T}}$ and we will have answer A with the string *ς*_*τ*_(*F*).

**Isomorphism.** Given a string *ς*_2_(*x*), if it is not in the list $V_{G_{2}}$, it terminates ⊥. Otherwise, if *x* already exists in the list $V_{G_{1}}$, it returns *ς*_1_(*x*) to A. If not, B sets a random string *ς*_1_(*x*) in {0,1}* that is distinct from any existing list $V_{G_{1}}$. Finally, B adds 〈*x*,*ς*_1_(*x*)〉 to $V_{G_{1}}$, and sets *ς*_1_(*x*). It then returns to A.

**Bilinear pairing.** Given two operations *ς*_1_(*x*),*ς*_2_(*y*), if *ς*_1_(*x*) not in the list $V_{G_{1}}$ and *ς*_2_(*y*) not in the list $V_{G_{2}}$, it terminates ⊥. Otherwise, B calculates *F* = *xy* mod *p* also checks if *F* in the list $V_{G_{T}}$. In that case, B returns to *ς*_*T*_(*F*). Otherwise, B sets a random *ς*_*T*_(*F*) in {0,1}* different from any existing $V_{G_{T}}$. Finally, B will add 〈*F*,*ς*_*T*_(*F*)〉 to the $V_{G_{T}}$ and reply A with string *ς*_*T*_(*F*).

Based on the basic operations of the above group, the simulation selects the security game as follows:

**Establishment.** Adversary A selects two different challenges with access control policies *P*_0_,*P*_1_. Here, *P*_*i*_ = {*P*_*i*,1_,*P*_*i*,2_,…,*P*_*i*,*n*_} where *i*∈{0,1}, and sends them to B.B does not select the true value for the variables of the master key $\left(\alpha ,\beta ,b,{\left\{{\left\{a_{i,j}\right\}}_{1\leq j\leq n_{i}}\right\}}_{1\leq i\leq n}\right)$, and just maintains it in the corresponding list. Then, B updates the list by adding a random string representation of tuples $\left(e{\left(g_{1},g_{2}\right)}^{\alpha },g_{1}^{b},g_{2}^{\alpha +\beta /b},e{\left(g_{1},g_{2}\right)}^{\alpha r},g_{1}^{br},r,{\left\{{\left\{g_{1}^{a_{i,j}}\right\}}_{1\leq j\leq n_{i}}\right\}}_{1\leq i\leq n}\right)$ consistent with each common parameter. Finally, B sets up a new update list to A.

Phase1. A selects a list of attributes $L=\left\{L_{1},L_{2},\cdots ,L_{n}\right\}=\left\{v_{1,t_{1}},v_{2,t_{2}}\cdots ,v_{n,t_{n}}\right\}$. For *O*_*KeyGen*_(*L*),*O*_*GenToken*_(*L*,*w*) queries, the premise is that A cannot ask the private key and token that satisfies the attributes of the access structure. The process is as follows:

*O*_*KeyGen*_(*L*):First, B uses 〈*α*,*ς*_*T*_(*α*)〉 instead of *e*(*g*_1_,*g*_2_)^*α*^. It adds new tuples 〈*αx*_*n*_,*ς*_*T*_(*αx*_*n*_)〉 of $e{\left(g_{1},g_{2}\right)}^{\alpha x_{u}}$ by the rules defined above, using variables *x*_*u*_ to the list $L_{G_{T}}$.Next, B increasing tuples $\left(x_{u},{\left\{{\left\{g_{2}^{\beta +a_{i,t_{i}}\lambda_{i}},g_{2}^{\lambda_{i}}\right\}}_{1\leq j\leq n_{i}}\right\}}_{1\leq i\leq n}\right)$ to update the list consistent with the private key, where *β*,*λ*_*i*_ is the new variable.

*O*_*GenToken*_(*L*,*w*):B runs *O*_*KeyGen*_(*L*).The list is then updated by adding tuples $\left(x_{u}+s,g_{2}^{\left(\alpha +\beta \right)s/b},{\left\{g_{2}^{\left(\beta +a_{i,t_{i}}\lambda_{i}\right)s},g_{2}^{\lambda_{i}H\left(w\right)s}\right\}}_{1\leq i\leq n}\right)$ consistent with the search token. *s* is the new variable.

Challenge: A chooses two keywords *w*_0_,*w*_1_. It then inputs 〈*w*_0_,*P*_0_〉,〈*w*_1_,*P*_1_〉 in the real choice of security games. The challenger chooses $\sigma \overset{R}{\leftarrow }\left\{0,1\right\}$ to encrypt *w*_*σ*_. Using *P*_*σ*_, the challenge index ciphertext of B is as follows: $\left({\left\{I_{i,1},{\left\{I_{i,j,2}\right\}}_{1\leq j\leq n_{i}}\right\}}_{1\leq i\leq n}\right)$.

For {*I*_*i*,1_}_1≤*i*≤*n*_, B adds tuples 〈*r*_*i*_,*ς*_1_(*r*_*i*_)〉 to the list $V_{G_{1}}$, and the new variable *r*_*i*_ satisfies $r=\sum_{i=1}^{n}r_{i}$.

For ${\left\{{\left\{I_{i,j,2}\right\}}_{1\leq j\leq n_{i}}\right\}}_{1\leq i\leq n}$,if *w*_0_ = *w*_1_ and $v_{i,t_{i}}\in P_{0,i}\wedge v_{i,t_{i}}\in P_{1,i}$, B adds tuples 〈*a*_*i*,*j*_*r*_*i*_/*H*(*w*),*ς*_1_(*a*_*i*,*j*_*r*_*i*_/*H*(*w*))〉 to list $V_{G_{1}}$.Otherwise, if $v_{i,t_{i}}\notin P_{0,i}\wedge v_{i,t_{i}}\notin P_{1,i}$, B adds tuples 〈*r*_*i*,*j*_,*ς*_1_(*r*_*i*,*j*_)〉 to list $V_{G_{1}}$ with a new variable *r*_*i*,*j*_.If $v_{i,t_{i}}\notin P_{0,i}\wedge v_{i,t_{i}}\in P_{1,i}$ or $v_{i,t_{i}}\in P_{0,i}\wedge v_{i,t_{i}}\notin P_{1,i}$, B increases tuples 〈*θ*,*ς*_1_(*θ*)〉 with a new variable *θ* to the list $V_{G_{1}}$.

Phase2. A repeats phase 1 of the inquiry. The requirement is that if *w*_0_ ≠ *w*_1_, A cannot ask *O*_*KeyGen*_(*L*),*O*_*GenToken*_(*L*,*ω*) when *L*| = *P*_0_∧*L*| = *P*_1_.

After making at most *q* queries, A terminates and returns to guessing *σ*′∈{0,1}. At this point, B selects a random value of $\sigma \overset{R}{\leftarrow }\left\{0,1\right\}$ and obtains the real challenge ciphertext. In list $V_{G_{1}}$, $g_{1}^{\theta }$ is replaced by $g_{1}^{a_{i,j}r_{i}/H\left(w_{\sigma }\right)}$.Finally, B returns a list of all the updated tuples to A.

Next, a detailed analysis of the B simulation is presented. The simulation of B is perfect if and only if no unexpected collisions occur. The so-called collision is for two different polynomials *F*_*τ*,*l*_,*F*_*τ*,*l*′_(*τ*∈{1,2,*T*}).For some *l*,*l*′, the corresponding random coding string of the difference cannot equal 0. Therefore, *F*_*τ*,*l*_−*F*_*τ*,*l*′_ = 0, and this unexpected collision occurs in the following two conditions.

In front of the replacement, on this occasion, we use theorem [37, 38].The probability of a collision occurring in list $V_{G_{1}},V_{G_{2}},V_{G_{T}}$ is expected to be *O*(*q*^2^/*p*) at most. For more details, refer to [37, 38].

After the replacement, it is proven that no new equations *F*_*k*,*l*_,*F*_*k*,*l*′_ can be created between polynomials after simulation, even if B is replaced by *a*_*i*,*j*_*r*_*i*_/*H*(*w*_*σ*_) for *θ*. We must note that an adversary cannot construct a query for a nonzero *F* = *F*_*k*,*l*_−*F*_*k*,*l*′_. It only occurs after substitution when *F* = 0. In an alternative security game, the adversary tries to distinguish $g_{1}^{a_{i,j}r_{i}/H\left(w_{0}\right)},g_{1}^{a_{i,j}r_{i}/H\left(w_{1}\right)}$ between two different keyword *w*_0_,*w*_1_ queries. Given $\delta_{1}\overset{R}{\leftarrow }Z_{p}$, the probability of distinguishing $g_{1}^{a_{i,j}r_{i}/H\left(w_{0}\right)}$ and $g_{1}^{\delta_{1}}$ is half the probability for adversary A to distinguish $g_{1}^{a_{i,j}r_{i}/H\left(w_{0}\right)}\;and\;g_{1}^{a_{i,j}r_{i}/H\left(w_{1}\right)}$.

As a result, we revise the game in order to determine whether A would be able to structure the queries of $e{\left(g_{1},g_{2}\right)}^{\gamma a_{i,j}r_{i}}$ for some $g_{2}^{\gamma }$.Then, it can distinguish $g_{1}^{a_{i,j}r_{i}/H\left(w_{0}\right)}$ and $g_{1}^{\delta_{1}}$. We prove that A cannot structure the queries for $e{\left(g_{1},g_{2}\right)}^{\gamma a_{i,j}r_{i}}$.

To construct *a*_*i*,*j*_*r*_*i*_, *a*_*i*,*j*_*r*_*i*_ is from *a*_*i*,*j*_*r*_*i*_/*H*(*w*_*σ*_) according to the simulation. When B replaces *θ* with *a*_*i*,*j*_*r*_*i*_/*H*(*w*_*σ*_), since *w*_0_ ≠ *w*_1_, it cannot obtain the search tokens that satisfy *L*| = *P*_0_∧*L*| = *P*_1_. Therefore, even if B submits a true value *a*_*i*,*j*_*r*_*i*_/*H*(*w*_*σ*_) to *θ*, it cannot eliminate *a*_*i*,*j*_*r*_*i*_. We then have, as follows.

Fix any *a*_*i*,*j*_*r*_*i*_ that arises after B’s replacement. We make the assumption that A can construct a query for *e*(*g*_1_,*g*_2_)^*v*^ where *v* is a non-zero polynomial containing *θ*, which also turns into zero after B replaces *a*_*i*,*j*_*r*_*i*_ for *θ*.

To construct such a *v*, A must cancel *a*_*i*,*j*_*r*_*i*_ in *v*. To our knowledge, there may be a different attribute value *v*_*i*,*j*′_(*j*′ ≠ *j*) of *L*_*i*_ in the access policy. Adversary A is able to get the ciphertext $g_{1}^{a_{i,j^{\prime }}r_{i}}$ of *v*_*i*,*j*′_(*j*′ ≠ *j*). Therefore, adversary A can obtain *a*_*i*,*j*_*r*_*i*_ in two ways. One is by pairing $g_{1}^{a_{i,j^{\prime }}r_{i}},g_{2}^{\beta +a_{i,j}\lambda_{i}}$, and the other is by pairing $g_{1}^{r_{i}},g_{2}^{\beta +a_{i,j}\lambda_{i}}$.

If adversary A pairs $g_{1}^{a_{i,j^{\prime }}r_{i}},g_{2}^{\beta +a_{i,j}\lambda_{i}}$, then adversary A needs to get information about $g_{2}^{a_{i,j^{\prime }}\lambda_{i}}$. However, in the entire simulation, the user knows $g_{2}^{a_{i,j}\lambda_{i}}$ and does not know $g_{2}^{a_{i,j^{\prime }}\lambda_{i}}$.Therefore, $g_{2}^{a_{i,j^{\prime }}\lambda_{i}}$ does not exist. In other words, it cannot be paired, so this situation cannot be achieved.

If adversary A pairs $g_{1}^{r_{i}},g_{2}^{\beta +a_{i,j}\lambda_{i}}$, A will obtain combination *βr*_*i*_+*a*_*i*,*j*_*r*_*i*_*λ*_*i*_ of the query. Adversary A wants to know *ρ*′*a*_*i*,*j*_*r*_*i*_. A new variable *ρ*″ is introduced, making *ρ*′ = *λ*_*i*_*ρ*″. First, A needs to structure *p*″*βr*_*i*_. As previously known, $r=\sum_{r=1}^{n}r_{i}$.It can be converted into the construct *p*″*βr*. In the entire simulation, the only way to ask A to construct *p*″*βr* is to combine *T*_0_,*I*_0_.$g_{2}^{\frac{\alpha +\beta }{b}s},g_{1}^{br}$ are used to get the query of the combination *αrs*+*βrs*. We need *βrs*, so to eliminate *αrs*, A combines $\hat{I},\tilde{t}$. *e*(*g*_1_,*g*_2_)^*αr*^,*x*_*u*_+*s* are used to get tuples *αr*(*x*_*u*_+*s*). Next, A uses −*αrx*_*u*_ to eliminate *αrx*_*u*_ and obtain *αrs*. Thus A obtains the required *βrs*. A new variable *ρ*‴ is introduced to make the *ρ*″ = *ρ*‴*s*. By asking for *p*‴*s*(*β*(*r*−∑_*i*′ ≠ *i*_*r*_*i*′_)), you can get *a*_*i*,*j*_*r*_*i*_. However, A cannot construct such an inquiry. The reasons are as follows.

Since *s* is randomly selected by the user, adversary A does not know its value. Hence, adversary A cannot find a *ρ*‴ to satisfy *ρ*″ = *ρ*‴*s*. Therefore, *ρ*‴ is not observed. According to the simulation challenge ciphertext, *I*_*i*,*j*,2_ has $v_{i,t_{i}}\notin P_{b,i}\wedge v_{i,t_{i}}\in P_{1-b,i}$. In other words, adversary A cannot obtain the private key *sk* and search token for search operations. Here, at least one of the $r_{i}^{\prime }$ is unknown, according to [6]. Since it is less than $\sum_{i^{\prime }\neq i}r_{i}^{\prime }$, we cannot get the query about *ρ*‴*s*(*β*(*r*−∑_*i′ ≠ i*_*r*_*i*′_)).

Then, it is proven that the encrypted message is secure and still operates in a general group model. The above theorem 1 is used to prove that the ciphertext is not distinguishable under the same access policy.

**Theorem 2.** Under the condition of Theorem 1, the adversary has the advantage over the ciphertext in the scheme as *O*(*q*^2^/*p*).

Proof: The establishment of the system and the basic operations of the group are similar to that in the proof of theorem 1. Therefore, they are not repeated in this study. In the system setup, the attacker chooses the policy that will attack P*.

Using the above same group, we use *ς*_1_(1) for *g*_1_, *ς*_2_(1) for *g*_2_, and *ς*_*T*_(1) for *e*(*g*_1_,*g*_2_). At the start of the build phase, the simulator randomly selects *α*,*b* from ${\mathbb{Z}}^{*}{}_{p}$.The public parameters $\left(e{\left(g_{1},g_{2}\right)}^{\alpha },g_{1}^{b},g_{2}^{\left(\alpha +\beta \right)/b},e{\left(g_{1},g_{2}\right)}^{\alpha r},g_{1}^{br},r,{\left\{{\left\{g_{1}^{a_{i,j}}\right\}}_{1\leq j\leq n_{i}}\right\}}_{1\leq i\leq n}\right)$ are sent to the adversary.

Query for private key *O*_*KeyGen*_(*L*): The premise is that A cannot ask for the private key of *L* that satisfies the access structure P*.

*O*_*KeyGen*_(*L*):First, B substitutes *e*(*g*_1_,*g*_2_)^*α*^ with 〈*α*,*ς*_*T*_(*α*)〉 and adds new tuples 〈*αx*_*n*_,*ς*_*T*_(*αx*_*n*_)〉 of $e{\left(g_{1},g_{2}\right)}^{\alpha x_{u}}$ using the rules defined above and using variables *x*_*u*_ from the list $L_{G_{T}}$. Then, B adds tuples $\left(x_{u},{\left\{{\left\{g_{2}^{\beta +a_{i,t_{i}}\lambda_{i}},g_{2}^{\lambda_{i}}\right\}}_{1\leq j\leq n_{i}}\right\}}_{1\leq i\leq n}\right)$ to update the list relevant to the private key, and *β*,*λ*_*i*_ are the new variables.

Ciphertext challenge: A selects two messages *m*_0_,*m*_1_ in the actual choice safety game. Challenger B selects $\sigma \overset{R}{\leftarrow }\left\{0,1\right\}$ to encrypt *m*_*σ*_ using P*.The challenge ciphertext as follows.

The simulation starts by selecting a random $\hat{r}$, setting *λ*_*i*_ for each of the relevant attributes, and *λ*_*i*_ for the random selection in ${\mathbb{Z}}_{p}$. The simulation randomly selects a *θ*. The constructed ciphertext is as follows: $\hat{c}=e{\left(g_{1},g_{2}\right)}^{\theta }$, ${\hat{c}}_{0}=B^{\hat{r}}$, ${\hat{c}}_{i,1}=g_{1}^{{\hat{r}}_{i}}$, and ${\hat{c}}_{i,j}=A_{i,j}^{{\hat{r}}_{i}}=g_{1}^{a_{i,j}{\hat{r}}_{i}}$. The challenge ciphertext is sent to the adversary.

For up to *q* times after the inquiry, A terminates and returns a guess *σ*′∈{0,1} of *σ*. Next, B chooses a random value of $\sigma \overset{R}{\leftarrow }\left\{0,1\right\}$, and obtains the real challenge ciphertext in list $V_{G_{1}}$ by $\hat{c}=m_{b}e{\left(g_{1},g_{2}\right)}^{\alpha \hat{r}}$ instead of $\hat{c}=e{\left(g_{1},g_{2}\right)}^{\theta }$.Finally, B returns to the list of all tuples to update A.

The challenger's task is to distinguish ciphertext $\hat{c}$ as $m_{0}e{\left(g_{1},g_{2}\right)}^{\alpha \hat{r}}$ or $m_{1}e{\left(g_{1},g_{2}\right)}^{\alpha \hat{r}}$.We now modify the game to distinguish $e{\left(g,g\right)}^{\alpha \hat{r}}$ from *e*(*g*,*g*)^*θ*^. Here, *θ* is randomly selected from ${\mathbb{Z}}_{p}$. If the game is not modified, and assuming that the opponent has an *ε* advantage, then, in the modified game, any adversary has at least an *ε*/2 advantage. It can be seen in two cases. One is that the adversary must distinguish $m_{0}e{\left(g_{1},g_{2}\right)}^{\alpha \hat{r}}$ from *e*(*g*_1_,*g*_2_)^*θ*^, and the other is to distinguish between $m_{1}e{\left(g_{1},g_{2}\right)}^{\alpha \hat{r}}$ and *e*(*g*_1_,*g*_2_)^*θ*^. It is obvious that the probabilities of the two are equal. We need to calculate the advantage that the adversary wins the game in the modified game.

Next, a detailed analysis of B is given. We note that B's simulation is perfect if there is no unexpected collision. The collision is for two different polynomials of *F*_*τ*,*l*_,*F*_*τ*,*l*′_(*τ*∈{1,2,*T*}) for some *l*,*l*′, and all the random strings that encode the corresponding difference are not equal to 0. Therefore, *F*_*τ*,*l*_−*F*_*τ*,*l*′_ = 0.This unexpected collision occurs in the following two situations:

Before the substitution. In this scenario, using theorem [37,38], the probability of an unexpected collision occurring in list $V_{G_{1}},V_{G_{2}},V_{G_{T}}$ is at most *O*(*q*^2^/*p*).

After the substitution. It is impossible to have a new equation that can be created between polynomials *F* = *F*_*k*,*l*_−*F*_*k*,*l*′_, even if B is replaced by $\alpha \overset{⌢}{r}$ for *θ* in the simulation. It is emphasized that adversary A cannot structure a query for a nonzero *F* = *F*_*k*,*l*_−*F*_*k*,*l*′_, and *F* = 0 after the substitution.

Note: If the adversary asks for the private key that satisfies the attributes of the access policy, the simulator does not give the appropriate private key. If the adversary already has the appropriate private key to access the structure, the game is terminated.

*θ* is only included in the *e*(*g*_1_,*g*_2_)^*θ*^ in $G_{T}$.We note that *F* = *F*_*k*,*l*_−*F*_*k*,*l*′_. If *F* = 0 after the substitution, then $F=\rho \alpha \hat{r}-\rho \theta$, where *ρ* is a constant. Note that *F* ≠ 0, $F+\rho \theta =\rho \alpha \hat{r}$. We can increase this inquiry to the artificial adversary. We will prove that the adversary cannot construct a query of $e{\left(g_{1},g_{2}\right)}^{\rho \alpha \hat{r}}$.

The only way the adversary can get $\alpha \hat{r}$ is through pair ${\hat{c}}_{0},k_{0}$.Since ${\hat{c}}_{0}=B^{\hat{r}}=g_{1}^{b\hat{r}},k_{0}=g_{2}^{\left(\alpha +\beta \right)/b}$ obtains $\alpha \hat{r}+\beta \hat{r}$, the adversary needs to eliminate $\beta \hat{r}$. To get $\beta \hat{r}$,the adversary needs to combine $K_{i,1},{\hat{c}}_{i,1}$ and $K_{i,2},{\hat{c}}_{i,2}$. Note that $K_{i,1}=g_{2}^{\beta +a_{i,t_{i}}\lambda_{i}},{\hat{c}}_{i,1}=g_{1}^{{\hat{r}}_{i}}$. Adversary A will get the $\beta {\hat{r}}_{i}+a_{i,t_{i}}\lambda_{i}{\hat{r}}_{i}$ query and wants to eliminate $\rho^{\prime }a_{i,t_{i}}{\hat{r}}_{i}$.As $K_{i,2}=g_{2}^{\lambda_{i}},{\hat{c}}_{i,2}=A_{i,j}^{{\hat{r}}_{i}}=g_{1}^{a_{i,t_{i}}{\hat{r}}_{i}}$, A will obtain $a_{i,t_{i}}{\hat{r}}_{i}\lambda_{i}$. We know that $\hat{r}=\sum_{r=1}^{n}{\hat{r}}_{i}$. However, we know that A cannot ask the private key of *L* that satisfies the access structure P*. Therefore, there exists a $a_{i^{\prime },t_{i}}{\hat{r}}_{i^{\prime }}\lambda_{i^{\prime }}$ that cannot be constructed in the above way since there are some attributes *i*′ that belong to *L*_*j*_ and *L* does not satisfy policy P*. Therefore, the adversary cannot get $\beta {\hat{r}}_{i^{\prime }}$.We know that $\hat{r}=\sum_{r=1}^{n}{\hat{r}}_{i}$; therefore, the adversary cannot obtain the value $\beta \hat{r}$.

## 6. Performance evaluation

The analysis of computational complexity: As our scheme is based on bilinear model, the computational complexity of the proposed scheme mainly comes from the pairing operation and group exponentiation operations in each group, ignoring all multiplication and hashing operations. The pairing operation is denoted by *P*. The group exponentiation operations in each group $G_{1},G_{2},G_{T}$ are represented by *E*_1_,*E*_2_,*E*_*T*_. The implementation uses the Pairing Based Cryptography (PBC) library[39]. The computational complexity of the proposed scheme with some existing latest similar schemes is analyzed in Table 1. In the scenario, we suppose that there are *n* attributes. The *i*-th attribute has *n*_*i*_ possible values such that *i* = 1,⋯,*n*.

**Table 1:** Computational complexity.

Algorithm	Computational complexity			
	[10]	[13]	[18]	Our scheme
Establish	3*E*_1_	$\left(\sum_{i=1}^{n}n_{i}+1\right)E_{1}+E_{T}$	3*E*_1_+*E*_*T*_	$\left(\sum_{i=1}^{n}n_{i}+2\right)E_{1}+E_{2}+2E_{T}$
KeyGen	(2*n*+2)*E*_1_	(2*n*+2)*E*_2_+*E*_*T*_	3*nE*_1_	2*nE*_2_+*E*_*T*_
Encrypted ciphertext	×	×	(3*n*+1)*E*_1_+(2*n*+1)*E*_*T*_	(2*n*+1)*E*_1_+*E*_*T*_
Encrypted index	(2*n*+3)*E*_1_	(2*n*+1)*E*_1_+2*E*_*T*_	×	2*nE*_1_
Token	(2*n*+2)*E*_1_	(2*n*+1)*E*_2_	×	(2*n*+1)*E*_2_
Search	(2*n*+2)*P*	(2*n*+1)*P*+*E*_*T*_	×	(2*n*+1)*P*+*E*_*T*_
Decrypt	×	×	2*nP*	(2*n*+1)*P*+*E*_*T*_

Functional analysis: A functional comparison of our scheme with some existing schemes is illustrated in Table 2. It includes hidden access structures, multi-data owners, encrypted messages, keyword search, and attribute changes, from which we can see that our scheme is fully functional.

**Table 2:** Functional comparison.

Scheme	Hidden policy	Multi owners	Encrypt		Update
Miao Y[10]	×	√	×	√	×
Yang K[12]	×	×	√	×	√
Qiu S [13]	√	×	×	√	×
Zhong H[18]	√	×	√	×	√
Our scheme	√	√	√	√	√

**Fig 2:**
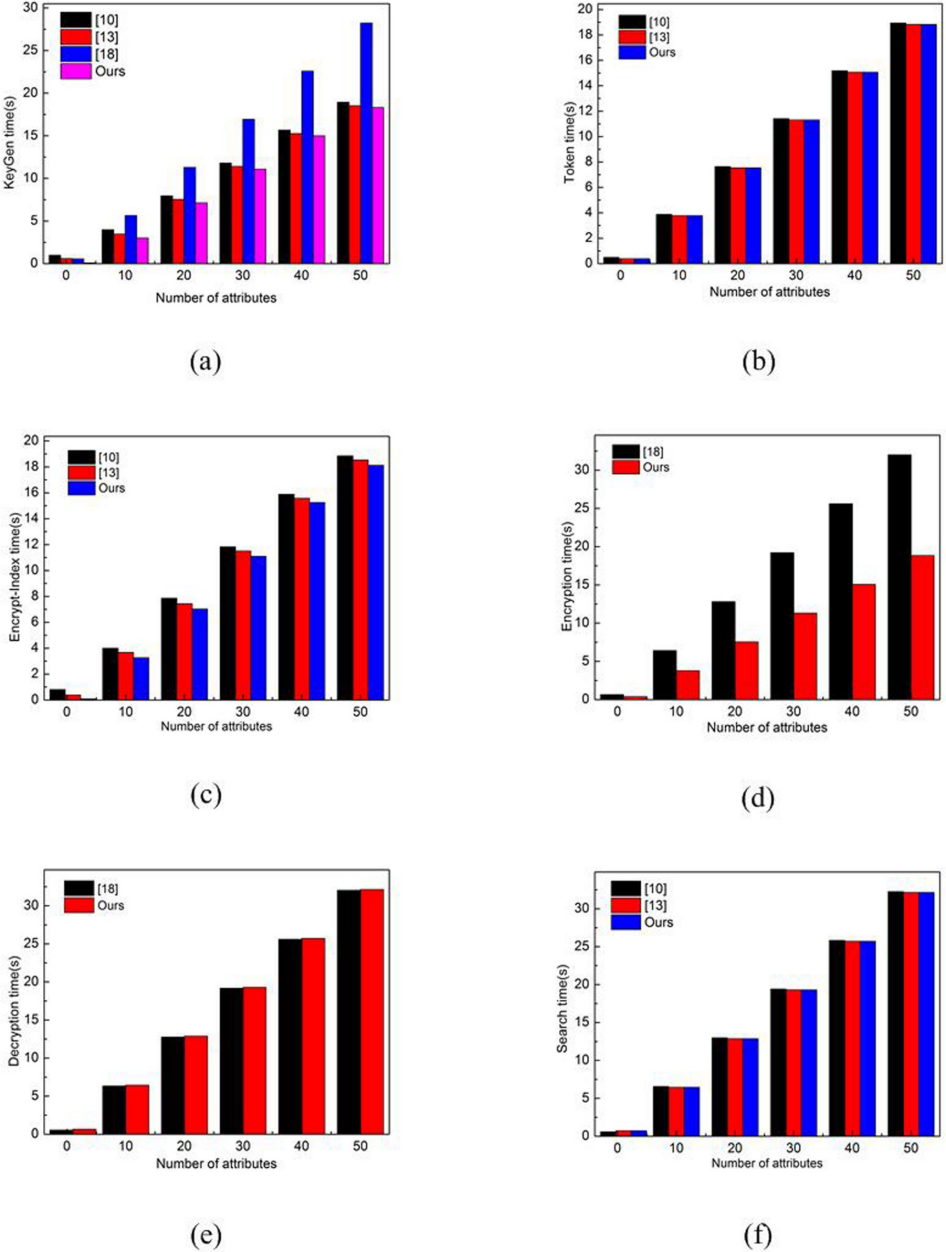
Computation comparison.

The actual analysis: The actual execution time of each algorithm in the simulation experiments are as follows. We let *n* range from 1 to 100 in the access structure where *n* is the number of involved attributes. *n*_*i*_ = 10, where *n*_*i*_ is the possible values of the *i*-th attribute. We list a comparison of the average computation time for each algorithm in the scheme with the algorithm in [10, 13, 18] in Fig 2.

From the experimental results of computation comparison shown in Fig 2, we can see that as the increase of the number of attributes, the computation times of private key generating, encryption-index time generating and encryption ciphertext time are slightly better than these schemes [10,13,18]. In our scheme, the computation times of token time generating and keyword search are close to these schemes in [10,13,18].We notice that the decryption time of our scheme is a little more than that in [18], this is caused by the fact that our scheme is multi owners while the scheme in [18] is a single data owner. The scheme [18] cannot implement the search function, and the schemes [10,13] cannot achieve encryption ciphertext and decryption function. So our scheme is much practical than the schemes in [10,13,18].

## 7. Conclusions

In this paper, we present a new keyword searchable attribute-based encryption scheme with a hidden access strategy and attribute revocation. The encrypted ciphertext are outsourced to the cloud. The hidden strategy can better secure the users’ privacy. Our proposed scheme is a fully functional scheme that addressed the keyword search problem and the attribute updating problem. Theoretical analysis, complexity calculation and practical operations show that our scheme is effective and practical. Of course, the scheme also has several short comings. The security of the scheme is demonstrated under the general bilinear group model, and it would be considerably better in the standard model.

## Supporting information

S1 File The runtime of cryptographic operations. (DOCX)
